# DNA sequence templates adjacent nucleosome and ORC sites at gene amplification origins in *Drosophila*

**DOI:** 10.1093/nar/gkv766

**Published:** 2015-10-10

**Authors:** Jun Liu, Kurt Zimmer, Douglas B. Rusch, Neha Paranjape, Ram Podicheti, Haixu Tang, Brian R. Calvi

**Affiliations:** 1 Department of Biology, Indiana University, Bloomington, IN 47405, USA; 2 School of Informatics and Computing, Indiana University, Bloomington, IN 47405, USA; 3 Center for Genomics and Bioinformatics, Indiana University, Bloomington, IN 47405, USA

## Abstract

Eukaryotic origins of DNA replication are bound by the origin recognition complex (ORC), which scaffolds assembly of a pre-replicative complex (pre-RC) that is then activated to initiate replication. Both pre-RC assembly and activation are strongly influenced by developmental changes to the epigenome, but molecular mechanisms remain incompletely defined. We have been examining the activation of origins responsible for developmental gene amplification in *Drosophila*. At a specific time in oogenesis, somatic follicle cells transition from genomic replication to a locus-specific replication from six amplicon origins. Previous evidence indicated that these amplicon origins are activated by nucleosome acetylation, but how this affects origin chromatin is unknown. Here, we examine nucleosome position in follicle cells using micrococcal nuclease digestion with Ilumina sequencing. The results indicate that ORC binding sites and other essential origin sequences are nucleosome-depleted regions (NDRs). Nucleosome position at the amplicons was highly similar among developmental stages during which ORC is or is not bound, indicating that being an NDR is not sufficient to specify ORC binding. Importantly, the data suggest that nucleosomes and ORC have opposite preferences for DNA sequence and structure. We propose that nucleosome hyperacetylation promotes pre-RC assembly onto adjacent DNA sequences that are disfavored by nucleosomes but favored by ORC.

## INTRODUCTION

Eukaryotic cells rapidly duplicate their genome by initiating DNA replication at multiple origins. Defects in origin regulation can cause DNA damage, developmental abnormalities and cancer (1). In multicellular eukaryotes, the rules for how certain genomic loci are selected to be active origins remain incompletely defined. Origin activity can differ among cells in development, which correlates with changes to the epigenome, but mechanisms are poorly understood (2). In this study, we investigate how nucleosome position influences the activity of origins responsible for developmental gene amplification in the *Drosophila* ovary.

Eukaryotic origins are binding sites for a multi-subunit pre-replicative complex (pre-RC) (3). During pre-RC assembly, origin DNA is first bound by the six subunit origin recognition complex (ORC). The ORC then recruits Cdc6 and Cdt1, which are required to clamp the hexameric MCM helicase around DNA to complete pre-RC assembly (3–10). The pre-RC assembles during G1 phase of the cell cycle, and is then activated by Cyclin / CDKs and Dbf4 / CDC7 kinases to initiate DNA replication during the subsequent S phase (10,11).

In multicellular eukaryotes, the pre-RC assembles and DNA replication initiates at preferred sites, but much remains unknown about how genomic loci are selected to be active origins. ORC has little DNA sequence specificity *in vitro* beyond a preference for AT-rich and negatively supercoiled DNA (12–18). Although a number of DNA sequence attributes have been reported to correlate with ORC binding sites and active origins, none of these genome-wide correlations are perfect (19–21). What is clear is that chromatin exerts a major influence over origins, and can result in different origin activities in different cell types (2,22). During development, changes to the epigenome influence where pre-RCs assemble, when during S phase they initiate replication (origin timing), and the fraction of cell cycles during which they do so (origin efficiency) (2,16,23–31). In general, origins that reside within active epigenome domains tend to initiate efficiently and early in S phase, whereas origins within heterochromatic domains are less efficient and initiate later in S phase, although there are exceptions to these rules (31–41).

While it is clear that chromatin impacts origin activity during development, the molecular mechanisms are incompletely defined. One common attribute among eukaryotic origins is that they correspond to nucleosome depleted regions (NDRs) (2,36,42–47). In fact, early evidence in yeast indicated that forcing a nucleosome over an origin inhibits its function (48). The observation that most origins are NDRs, together with the promiscuous DNA binding of ORC *in vitro*, suggested that ORC binds DNA *in vivo* wherever it is not occluded by nucleosomes. In contrast, more recent evidence suggests that nucleosomes adjacent to some origins may actually play a positive role in promoting ORC binding and origin activation at select sites (46,49–50). Specific modifications of nucleosomes at origins promote pre-RC assembly and activation, including acetylation and methylation of specific histone lysines (51–61). Conversely, evidence suggests that heterochromatin inhibits origins by hindering recruitment of proteins required for replication initiation (62). Despite these important advances, these nucleosome modifications are not instructive at all origins, and much remains to be learned about how chromatin influences pre-RC assembly and activation.

**Figure 1.**
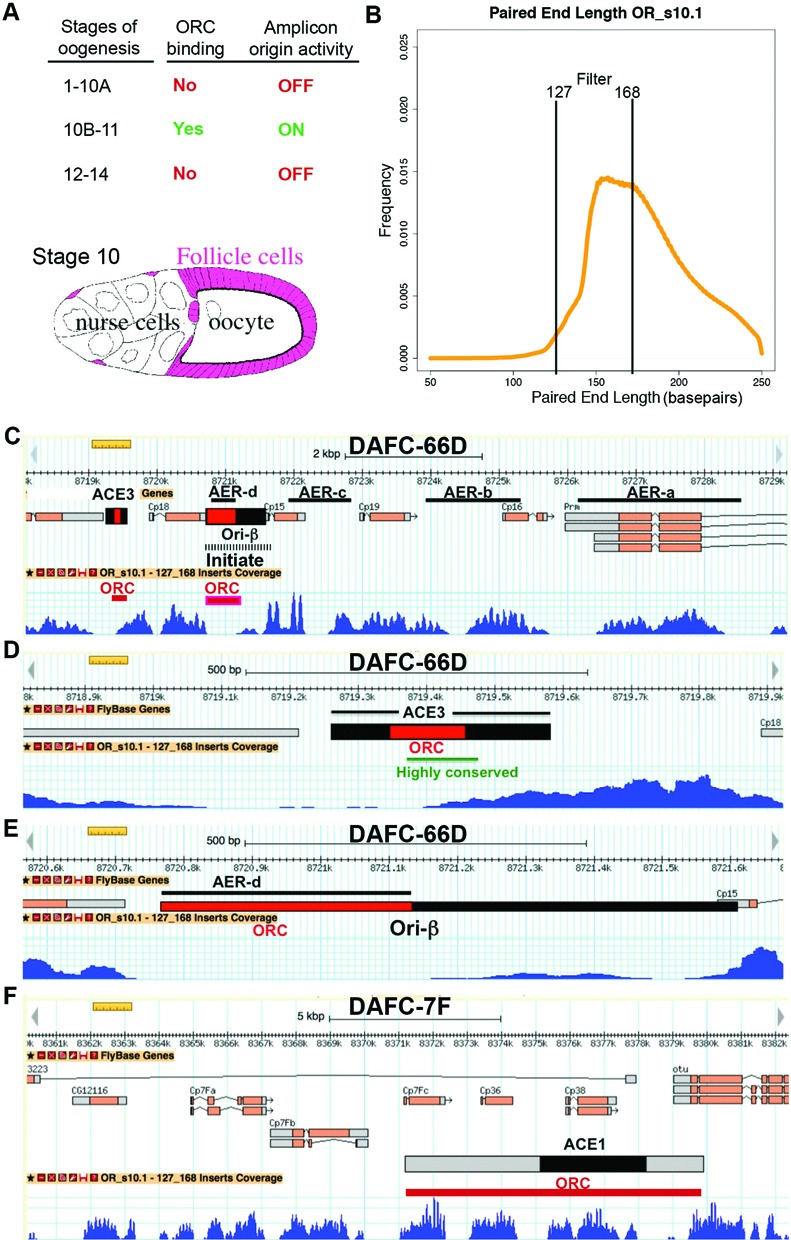
Sequences important for amplicon function are depleted of nucleosomes in stage 10 follicle cells. (**A**) Amplicon origin activity is restricted to follicle cells during late oogenesis. DAFC-66D is bound by ORC and active specifically during stages 10B-11 of oogenesis. Below is a stage 10 egg chamber showing the location of the somatic follicle cells (pink) that surround the germline oocyte and nurse cells. (**B**) Size distribution of all DNA fragments after MNase digestion of stage 10 follicle cell genomic DNA from *Oregon-R* (OR_s10.1) as determined by paired end sequencing. The filtered size range of 127–168 was used to map nucleosome positions. (**C**–**F**) Nucleosome positions at the two major amplicons on the 3^rd^ (DAFC-66D) and X (DAFC-7F) chromosomes. In each panel, the genomic coordinates and organization of the amplicons are shown above, and the corresponding signal graph for nucleosome occupancy shown in blue below. Transcription units are indicated by boxes, with translated regions in orange. Each locus has multiple chorion protein (cp) genes. Preferred initiation sites are indicated by black hashed lines and preferred ORC binding sites as red lines, but both amplicons reside within a larger zone of distributed ORC binding. (**C**) Nucleosome position at DAFC-66D during stage 10. Amplification enhancing elements (AERs a–d) stimulate origin activity and were defined by deletion mapping (87). The ACE3 and Ori-β are essential for origin activity, with the regions most important for origin function that correspond to ORC binding sites (red lines). (**D**) Enlargement of the ACE3 region. The green line demarcates an A-T rich region in ACE3 that is highly conserved at the amplicon in the genus *Drosophila*. (**E**) Enlargement of Ori-β region, the preferred site of replication initiation. (**F**) Nucleosome position at DAFC-7F. ACE1 is important for origin function, binds ORC, and is shown as a box, with the central region that has the strongest effect on origin function shown as a black box.

To understand how chromatin regulates origins, we have been studying developmental gene amplification during *Drosophila* oogenesis. During gene amplification, origins at six genomic loci repeatedly initiate DNA replication, which results in a local increase in the copy number of genes required for eggshell (chorion) synthesis (23,63–66). These amplicon origins are bound by the pre-RC and active in ovarian follicle cells only during late oogenesis, a time when other origins are not active and genomic replication has ceased (Figure 1A) (67–70). We and others previously showed that the acetylation of nucleosomes at amplicon origins during late oogenesis promotes ORC binding and replication initiation (71,72). Origin nucleosomes are hyperacetylated on multiple histone lysines specifically during stages 10–12 of oogenesis when ORC is bound and the origins are active, followed by a rapid deacetylation in stage 12 that correlates with the departure of ORC and shut off of the amplicon origins (66,68–70). The region of highest nucleosome acetylation corresponds to preferred ORC binding sites, but there is a diminishing gradient of nucleosome acetylation and ORC binding that extends outwards from the origins over an ∼15–20 kb epigenome domain (66,70). Evidence suggests that multiple histone acetyl-transferases (HATs) acetylate different histone lysines to promote different steps of ORC binding and pre-RC assembly (70–71,73). The recruitment of these chromatin modifiers to the amplicon origins may be mediated in part by a transcription factor complex known as Myb-MuvB (MMB) (74,75). While evidence strongly supports a role for nucleosome acetylation in amplicon origin activity, an understanding of molecular mechanism is far from complete.

In this study, we used paired-end illumina sequencing to determine how nucleosome position influences the developmental specificity of the amplicon origins. We focus on *Drosophila* amplicon in follicle cells at 66D (DAFC-66D) at which ORC binding sites and other sequences important for origin function have been mapped to high resolution. Similar to other origins, ORC binding sites at DAFC-66D are depleted of nucleosomes. Further integrative analysis suggests that ORC binds to these NDRs not just because they are depleted of nucleosomes, but also because ORC and nucleosomes have different preferences for DNA sequence and structure. Our data lead us to propose that hyperacetylation of positioned nucleosomes promotes pre-RC assembly onto adjacent DNA sequences that are disfavored by nucleosomes but preferred by ORC. DAFC-66D may be a member of an emerging subclass of origins at which DNA sequence and nucleosome modification promotes pre-RC assembly.

## MATERIALS AND METHODS

### *Drosophila* strains

The *Oregon-R^modENCODE^* and *P{w^+mW.hs^ C323:GAL4}* strains were obtained from the Bloomington *Drosophila* Stock Center, (BDSC, Bloomington, IN). *P{w^+mW.hs^, UAS:dacapo*} was a gift from the Hariharan lab (76). All crosses were conducted at 25°C.

### Mass isolation and purification of follicle cell nuclei

Follicle cell nuclei were mass isolated and purified using methods as previously described (70). Briefly, well-fed, fertilized females were homogenized in a blender to release individual egg chambers. Egg chambers from three different stages of oogenesis (≤s8, s10, and s12–13) were purified by serial filtration through 250–70 μm meshes, fixed with 2% paraformaldehyde solution for 15 min at room temperature and then treated with 125 mM glycine to quench the fixation. Egg chambers were further purified by hand selection of desired stages in DPBS buffer under the dissecting microscope. Samples were stored at −80°C before proceeding to nuclear preparation. Frozen staged egg chambers were thawed on ice, re-suspended in 1 ml mHB buffer (0.34 M Sucrose, 15 mM NaCl, 60 mM KCl, 0.2 mM EDTA, 0.2 mM EGTA, 0.15 mM Spermine, 0.15 mM Spermidine in 15 mM Tris–HCl, pH 8.0) supplemented with 0.5% NP-40, and dounced 15 strokes in Kontes 2 ml douncer with type A pestle. The sample was then filtered through 15 μm Nytex nylon mesh to purify the follicle cell nuclei from the larger nurse cell nuclei. The filtrate was then centrifuged for 3 min at 500 x g to pellet follicle nuclei. Follicle cell nuclei were isolated from approximately 3000 egg chambers for each sample (≤s8, s10, and s12–13) and a comparable tissue volume were used in each experiment.

### Micrococcal nuclease sequencing

Follicle cell nuclei were resuspended in 100 μl digestion buffer (50 mM Tris–HCl, 5 mM CaCl_2_, pH 7.9) and digested with 50 U micrococcal nuclease (New England Biolabs, cat# M0247S) at 37°C for 20 min, quenched with 50 mM EGTA, followed by 2 μg RNaseA (Roche) treatment at 37°C for 1 h, and 40 μg proteinase K treatment at 55°C for 1 h. NaCl was then added to a final concentration of 250 mM to solubilize undigested DNA (i.e. nucleosome protected). The undigested DNA was purified by phenol/chloroform/isoamyl alchohol extraction, ethanol precipitated, and resuspended in 1× TE buffer. DNA fragments in the 100–200 bp size range were purified on a 1.6% agarose gel and recovered with Qiaquick gel purification kit (Qiagen). A bar-coded, paired-end library was constructed using the standard Illumina protocol and 51 bp paired-end sequences obtained at the Tufts University Core Facility Genomics (TUCF genomics, http://tucf-genomics.tufts.edu). A coverage of ≥30× was obtained for all samples analyzed.

### Extraction of inserts protected by single nucleosomes

Paired end reads from Tufts genomics sequencing center in FASTQ format were mapped to *Drosophila melanogaster* reference genome (Flybase version 5.43), using BWA version 0.6.1-r104. A maximum of 6% edit distance, which allows three mismatches per 51 nucleotide (n.t.) read, and a minimum mapping quality of 37 (BWA definition) were used in the mapping. A size filter of 127 to 168 n.t. was also applied to include single nucleosome reads before further analysis.

### Analysis of nucleosome and ORC positions

After mapping the reads to the genome, nucleosomal peaks were detected using nucleR, an R package for non-parametric nucleosome positioning (77). For preprocessing, the nucleR Fourier transform function was applied and the peaks were called with parameters of threshold >50% and score >0.25. Internucleosomal distance is then defined as the distance between any two adjacent called peaks by nucleR. A nucleosomal depleted region (NDR) is any internucleosomal region without any called nucleosomal peaks. ORC site data was derived from Orc2 ChIP-seq project by modENCODE, ID#2753 (78). Orc sites that were defined by that study as having a score ≥7.5 were used to generate the graphs in Figure 5. In Supplementary Figure S4, all ORC site scores were plotted against NDR size. The NDR regions in *Drosophila* S2 cells in culture were calculated from previously published MNase-Seq reads (79). These ORC and nucleosome positions were then used with in-house scripts to generate graphs in Figure 5 and Supplementary Figure S4.

### DNA sequence signatures of nucleosome and ORC sites

The prediction of nucleosome positions at DAFC-66D was determined in silico using a web interface from the Segal Lab: http://genie.weizmann.ac.il/software/nucleo_prediction.html.

Nucleotide composition at DAFC-66D was determined over a sliding 50 bp window using the nucleotide composition analysis software of MacVector (version 13.5.2) (80). Quantitative ORC binding to different DNA fragments and nucleosome acetylation data were from previously published sources (12,70).

#### Data access

The six follicle cell MNase-Seq data sets have been deposited into NCBI-SRA under BioProject SRP057811.

## RESULTS

### ORC binding sites and other sequences important for amplicon origin function are depleted of nucleosomes

To understand the relationship between nucleosomes and amplicon origin activity, we mapped nucleosome position in follicle cells to nucleotide resolution using micrococcal nuclease digestion followed by paired-end Illumina sequencing (MNase-Seq). We began by examining stage 10 egg chambers, a time in oogenesis when amplicon origins are first bound by ORC and initiate DNA replication (Figure 1A) (63,67,69). We used a wild type *Oregon-R* strain that was also used by the modENCODE project for genomic and epigenomic mapping (*OR^modENCODE^*) (81). To achieve a high developmental resolution, we used a mass isolation procedure to collect different stages of oogenesis (70). After fixation, stage 10 egg chambers were further hand selected and follicle cell nuclei were purified from larger nurse cell nuclei by filtration (Figure 1A)(70). Nuclear chromatin was digested with MNase, and the protected DNA fragments were isolated and subjected to paired-end illumina sequencing. Analysis of the sequencing reads revealed an ∼150 bp modal fragment size that was protected from MNase digestion, consistent with the known length of DNA wrapped around a nucleosome (Figure 1B). There was a distribution of smaller and larger fragments, which in other studies have been shown to result from partial wrapping of DNA around nucleosomes or protection from MNase digestion by other proteins (82–84). To analyze the fragment population that reflects protection by single nucleosomes, we filtered the data to a fragment size range of 127–168 bp, and then mapped these fragments to the annotated *Drosophila* genome (Figure 1B) (85,86).

We focused our analysis on the well-characterized DAFC-66D amplicon where ORC binding sites and functional *cis* sequences have been mapped to highest resolution. Mapping of MNase-Seq reads indicated that most nucleosomes at DAFC-66D occupied the exonic regions of the four chorion protein (cp) genes, whereas the introns and intergenic sequences were nucleosome depleted regions (NDRs) (Figure 1C). These intergenic NDRs overlapped amplification enhancer regions (AERs a–d), which contribute quantitatively to origin function (Figure 1C) (87,88). Importantly, NDRs corresponded to two regions that are essential for origin function; the 320 bp Amplification Control Element on 3 (ACE3) and the 840 bp Ori-β (Figure 1C–E). Both ACE3 and Ori-β are required for origin function, both are bound by ORC *in vitro* and *in vivo*, and Ori-β is the preferred replication initiation site (12,63,68,87–91). While the 5′ end of ACE3 was a NDR, its 3′ end overlapped a nucleosome occupied region (Figure 1C,D). The 3′ boundary of this NDR in ACE3 corresponded to a 72bp poly-A:T sequence that has been highly conserved among orthologous amplicons in the genus *Drosophila* over 40 million years of evolution (Figure 1D) (92–94). The ACE3 NDR also corresponds to regions bound by the Myb-MuvB transcription factor complex (74). Ori-β also corresponded to an extended region of very low nucleosome occupancy (>700 bp) (Figure 1C and E). The 5′ Ori-β subregion is crucial for origin function and was especially devoid of nucleosomes (Figure 1E) (12,68,90). Biological replicates of the MNase-Seq using independently-isolated stage 10 follicle cell nuclei for a new sequence run yielded a highly similar nucleosome occupancy map (Supplementary Figure S1A and B). Together, these results show that the ORC binding sites ACE3 and Ori-β and other regions important for DAFC-66D origin function have low nucleosome occupancy in stage 10 follicle cells.

To determine if low nucleosome occupancy is a property of regulatory elements at other amplicon origins, we examined the amplicon on the X chromosome, DAFC-7F, during stage 10 (95,96). Similar to DAFC-66D, at DAFC-7F exon regions were most occupied by nucleosomes, while the introns and intergenic regions among the four X-linked chorion protein genes were NDRs (Figure 1F). The region most important for DAFC-7F origin function is the ∼3 kb Amplification Control Element on 1 (ACE1), a region bound by ORC *in vitro* and *in vivo* (Figure 1F, black box) (68,96). The 5′ end of ACE1 is most critical for origin function and overlapped an NDR in the promoter region of the cp38 gene (Figure 1F) (96). A larger region spanning ACE1 that is known to further stimulate origin function corresponded to several NDRs (Figure1F gray box) (96). Biological replicates yielded highly similar results for nucleosome position at DAFC-7F (Supplementary Figure S1C). Thus, like DAFC-66D, it appears that some of the functional origin sequences at DAFC-7F are NDRs, while others are at least partially occupied by nucleosomes.

Given that the origin is actively initiating replication in stage 10, we considered the possibility that replication forks moving through the region may affect average nucleosome position. To address this, we repeated the MNase-Seq analysis on follicle cells in which replication initiation was blocked by expressing the CDK2 inhibitor *dacapo (dap)* (97,76). We had previously shown that expression of a *UAS:dacapo* transgene in follicle cells using the c323GAL4 driver strongly inhibits Cyclin E / CDK2 and completely blocks the initiation of DNA replication at the amplicons (67,70). Analysis of the MNase-Seq results from these egg chambers revealed that nucleosomes at DAFC-66D and DAFC-7D have similar positions in wild type and *dap* expressing follicle cells, indicating that the movement of replication forks through this region does not result in gross nucleosome repositioning at the active origins (Supplementary Figure S1A-C).

We also mapped nucleosomes at the four other DAFC loci that are amplified to lower levels (∼4–8 fold) in follicle cells (66–67,70,98–100). This showed that intergenic regions at these loci were also NDRs. (Supplementary Figure S2A-D). Previous nascent strand mapping showed that the replication initiation site at DAFC-34B occurs near the Vm34Ca gene, a site that is adjacent to an especially large NDR of >5 kb (100). At DAFC-62D, three ORC binding sites have been mapped that spanned regions that are both occupied and unoccupied by nucleosomes (Supplementary Figure S2D)(101). It was previously shown that activity of the DAFC-22B amplicon is strain-specific, and that it is neither active nor hyperacetylated in the *OR^modENCODE^* strain that we used for MNase-seq (66,70). Nevertheless, at DAFC-22B, there were NDRs that were comparable in size or larger than those at the other active DAFC loci (Supplementary Figure S2A). Thus, at the minor amplicons, some ORC binding sites and other sequences important for origin function are NDRs, while others are occupied by nucleosomes. It is important to note, however, that for those regions where ORC and nucleosomes map to the same site, it cannot be concluded that they bind simultaneously to the same DNA fiber because these data are a snapshot of average nucleosome position in a population of cells.

### NDR size does not correlate with ORC binding sites or active origins

**Figure 2.**
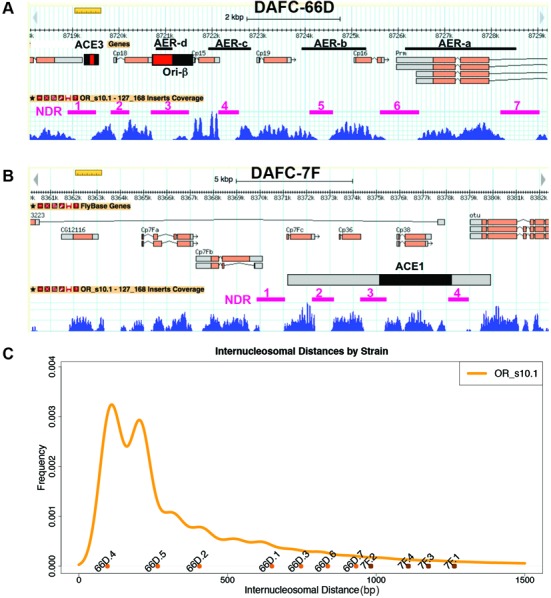
Nucleosome depleted regions (NDRs) at the amplicons are not exceptionally large. NDRs at DAFC-66D (**A**) and DAFC-7F (**B**) are demarcated by magenta lines and numbered. (**C**) Comparison of amplicon NDR sizes, defined by internucleosome distance, to the distribution of all NDRs genome-wide. The orange line represents the distribution of all NDR sizes in the follicle cell genome during stage 10. The dots represent the size of the different NDRs from DAFC-66D (66D) and DAFC-7F (7F) as defined in panels (A) and (B).

The data at DAFC-66D revealed a correlation between intergenic NDRs, ORC binding sites, and functional origin sequences. This result is consistent with evidence from yeast to mammals that most ORC binding sites and active origins are NDRs (2). At DAFC-22B, however, there were several NDRs, but this origin is not active in the *OR^modENCODE^* strain (66,70). To assess if NDR size correlates with origin activity, we compared the size of amplicon origin NDRs to the distribution of inter-nucleosome distances for the entire follicle cell genome (Figure 2A–C). The genome-wide NDR size distribution was bimodal with two prominent peaks at ∼120 and ∼200 bp, although there were many larger NDRs up to several kb in size, similar to previously published MNase-Seq data for NDR size distribution in cultured *Drosophila* cells (see below) (Figure 2C) (79). Some of the amplicon NDRs were larger than the modal inter-nucleosomal distance, for example the ∼700 bp NDR of Ori-β at DAFC-66D (Figure 2A-C)(66). Importantly, while many loci have NDRs that are larger than those at the amplicons, previous ORC ChIP-array data indicated that they are not bound by ORC in stage 10 follicle cells (66). These data suggest that large NDR size is not sufficient to specify an ORC binding site or active origin in stage 10 follicle cells.

### Nucleosome position does not change with amplicon origin activity

Nucleosomes at amplicon origins are hyperacetylated only during stages 10–12 of oogenesis, and this contributes to ORC binding and origin efficiency (66,69–72). It is known that the acetylation of nucleosomes at gene promoters can recruit ATP-dependent nucleosome remodeling complexes that reposition nucleosomes, thereby allowing transcription factors to gain access to their DNA binding sites (102). We wondered, therefore, whether the nucleosome acetylation at amplicon origins also alters nucleosome position, permitting ORC access to its binding sites in stage 10, and thereby controlling the developmental timing of origin activation.

**Figure 3.**
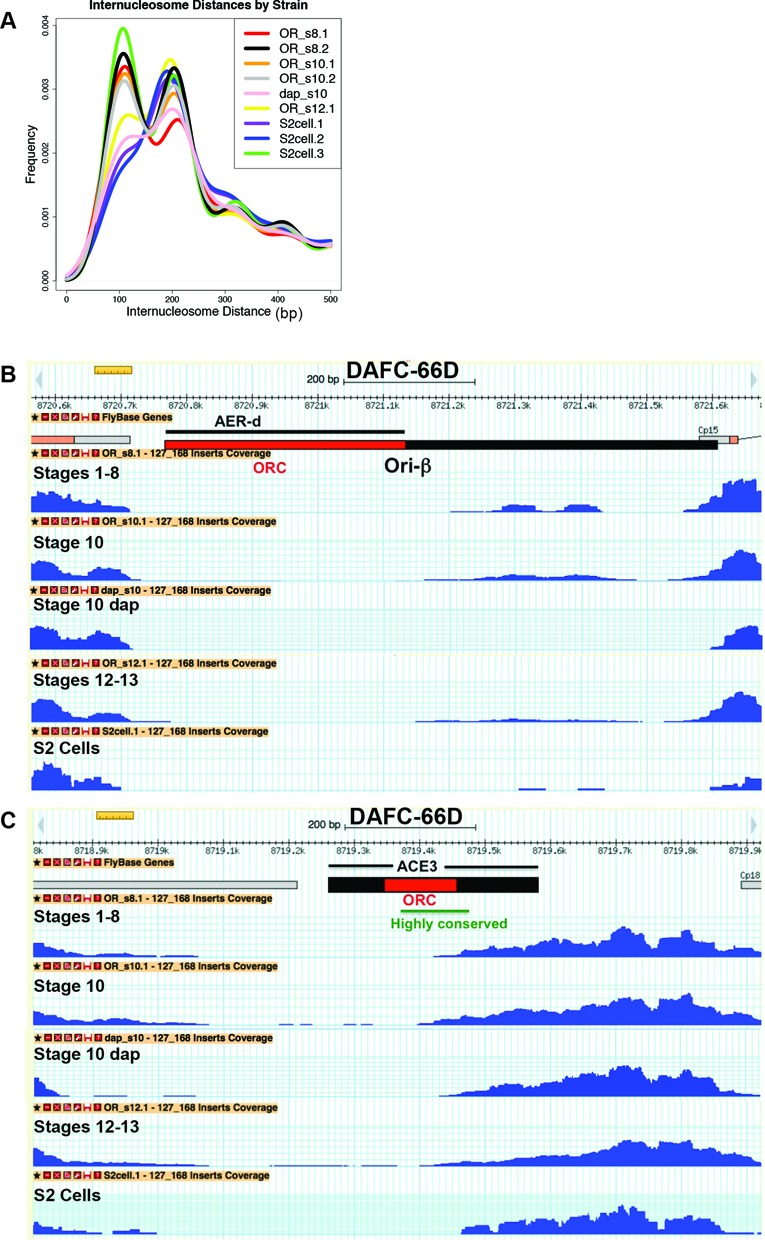
Nucleosome positions at DAFC-66D are similar among different stages and cell types. (**A**) Distribution of internucleosomal distances in Mnase-Seq data for follicle cells from different stages of oogenesis and embryonic S2 cells in culture. Follicle cells from stages 1–8 (OR_s8.1, OR_s8.2), stage 10 (OR_s10.1, OR_s10.2), or stage 12–13 (OR_s12.1), or stage 10 in which replication was inhibited by expressing dacapo (dap_s10). Numbers after the decimal points represent biological replicates. Internucleosomal distance for replicates of S2 cells in culture (S2cell.1, S2cell.2, S2cell.3) represents analysis of published MNase reads (79). (**B** and **C**) Nucleosome occupancy at DAFC-66D Ori-β (**B**) and ACE3 (**C**) are similar in follicle cells from different stages of oogenesis and S2 cultured cells.

**Figure 4.**
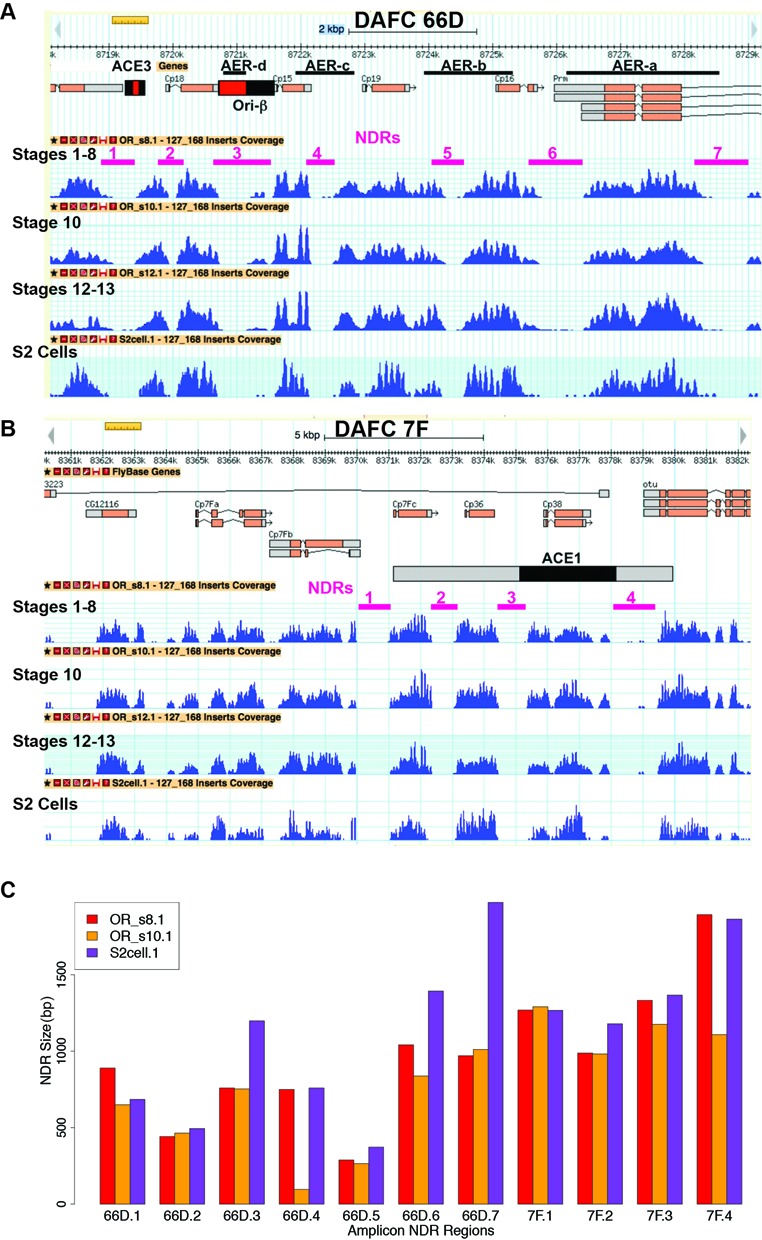
NDR sizes at DAFC-66D and DAFC-7F are similar among different stages and cell types. (**A** and **B**) Nucleosome occupancy in follicle cells during different stages of oogenesis and in S2 cells in culture at DAFC-66D (**A**) and DAFC-7F (**B**). The different NDRs at the origins are indicated by magenta lines and numbers. (**C**) Comparison of NDR sizes in follicle cells at stages 1–8 (OR_s8.1), stage 10 (OR_s10.1), and in S2 cultured cells (S2cell.1) (79). The different NDR's at DAFC-66D (66D) and DAFC-7F (7F) correspond to the numbers shown in (A) and (B).

To investigate this possibility, we used MNase-Seq to compare nucleosome position in follicle cells at stages of oogenesis before the amplicon origins are active (stages 1–8, hereafter ≤8), while the origins are active (stage 10) and after the origins have shut off (stages ≥12) (Figure 1A). We also compared nucleosome position in follicle cells with extant MNase-Seq data for *Drosophila* S2 cells in culture (embryo-derived), a cell type in which the amplicon origins are not active (Figure 3A) (44,79,103). This analysis revealed that nucleosome position at DAFC-66D and DAFC-7F are virtually identical among follicle cells during all stages of oogenesis and in S2 cells, including the NDRs corresponding to the ORC binding sites in ACE3 and Ori-β (Figures 3B, C, 4A–C). Analysis of the other four amplicons also showed that nucleosome position and NDR size is highly similar among different stages of oogenesis and in S2 cells (Supplementary Figure S3A–D). These data suggest that dynamic nucleosome repositioning does not govern the developmental specificity of ORC binding and amplicon origin activity.

### ORC NDRs are similar between S2 and follicle cells, but NDRs are not sufficient to predict ORC occupancy

**Figure 5.**
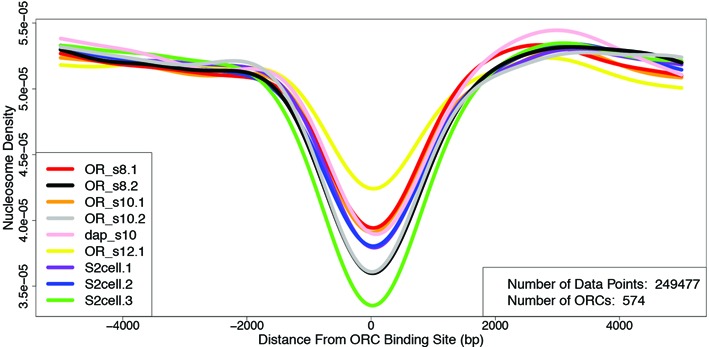
ORC binding sites are nucleosome depleted in S2 cells and ovarian follicle cells. Average nucleosome occupancy at ORC sites genome wide (y-axis) is plotted around the center of the ORC binding sites (x-axis) for follicle cells at different stages (OR_s8.1, OR_s8.2, OR_10.1, OR_10.2, dap_s10, OR_s12.1) and S2 cells in culture (S2cell.1, S2cell.2, S2cell.3). ORC sites with binding score >7.5 and S2 cell MNase-Seq data were from published sources (36,79).

The striking conservation of nucleosome position at amplicons between ovarian follicle cells and embryonic-derived S2 cells in culture prompted us to ask if nucleosome position is conserved between these cell types at other loci. To focus on the relationship of NDRs to origins, we analyzed the loci that bind ORC in cell culture (36). It was previously shown that these ORC binding sites are depleted of nucleosomes (36). We repeated this analysis using published ORC and nucleosome positions in S2 cells, focusing on significant ORC sites with a binding score of >7.5, as defined by previous studies (36,79). The results confirmed that ORC tends to bind in regions of the S2 cell genome that are relatively depleted of nucleosomes (Figure 5) (36). Analysis of our MNase-Seq data indicated that these same ORC sites are also depleted of nucleosomes in follicle cells during stages <8, 10 and 12-13 of oogenesis (Figure 5, Supplementary Figure S4). This includes stage 10 follicle cells in which only a subset of loci are bound by ORC, and in which only five loci are active origins. Thus, nucleosome depletion is not a strong predictor of ORC binding or active origins in stage 10 follicle cells. Moreover, within S2 cells there are many large NDRs that are not bound by ORC during genomic replication (36). Thus, while ORC has relaxed DNA binding specificity *in vitro*, and its binding sites strongly correlate with low nucleosome occupancy *in vivo*, ORC does not simply bind where DNA is not occupied by nucleosomes.

### Adjacent nucleosome and ORC positions at DAFC-66D are partially determined by DNA sequence

**Figure 6.**
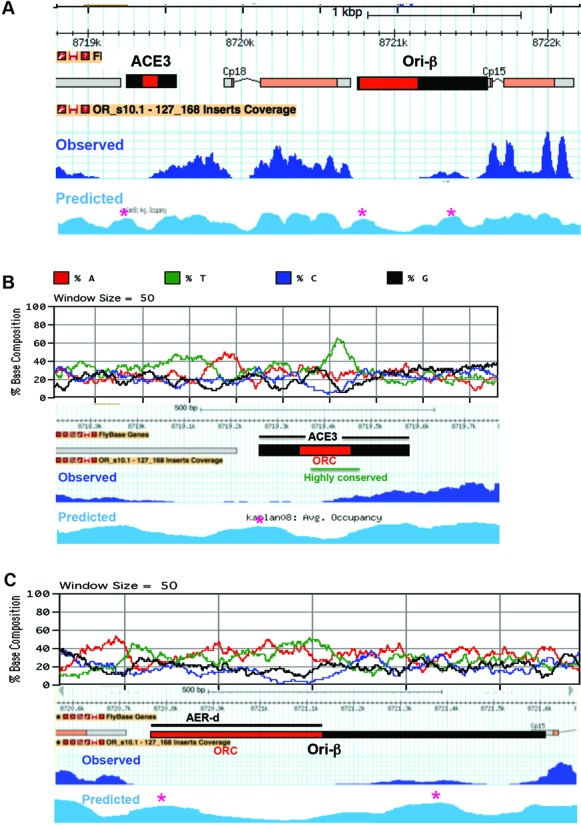
Nucleosome position at DAFC-66D is largely determined by primary DNA sequence. (**A**) Comparison of observed nucleosome occupancy at DAFC-66D in follicle cells in stage 10 (dark blue) with predicted nucleosome occupancy (light blue) based on nucleosome DNA sequence preferences (104,105). Red asterisks indicate three predicted nucleosome positions that were less occupied *in vivo* than predicted. (**B** and **C**) Expanded view of predicted and observed nucleosome occupancy at ACE3 (**B**) and Ori-β (**C**), with nucleotide composition plotted above (see color key). Nucleosome occupied sites are relatively GC rich (black and blue), while nucleosome depleted regions in ACE3 and Ori-β contain extended poly A:T tracts (red and green) that correspond to ORC binding sites.

It is known that nucleosomes prefer specific types of DNA primary sequence, but disfavor others, and that this is a major determinant of nucleosome position *in vivo* (104,105). Consistent with this, our data indicated that nucleosome position is highly similar among different stages and cell types genome wide (Figure 3A and data not shown). At other loci, however, there were differences in nucleosome position among stages and cell types, consistent with active nucleosome remodeling contributing to nucleosome position *in vivo* (106). To evaluate the contribution of DNA sequence to nucleosome position at DAFC-66D, we analyzed the locus using an algorithm that predicts nucleosome position based on the previously-determined sequence signatures for stable DNA-nucleosome interactions (104,105). We focused on the ∼3.8 kb minimal origin that spans ACE3 and Ori-β, which is sufficient to direct amplification when inserted at ectopic genomic sites (107,108). The nucleosome occupancy predicted from *in silico* analysis of DNA sequence was highly similar to the observed MNase-Seq nucleosome occupancy *in vivo*, with only three positions of notably different occupancy (Figure 6A-C). Specifically, there was a close match between predicted and observed nucleosome occupied sites over the cp18 and cp15 transcription units, as well as the region in between ACE3 and cp18. Similarly, there was a strong correspondence between DNA sequences predicted to be strongly disfavored by nucleosomes and the observed NDRs *in vivo*, including Ori-β and ACE3 (Figure 6A-C). These results suggest that while active nucleosome remodeling may contribute, DNA sequence has a major influence on nucleosome position at DAFC-66D.

**Figure 7.**
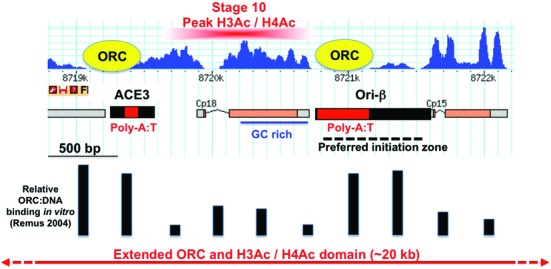
ORC binding to DAFC-66D is influenced by DNA sequence and hyperacetylation of positioned nucleosomes. The DAFC-66D 3.8 kb minimal origin with nucleosome occupancy *in vivo* (blue) shown above and relative ORC binding to different naked DNA fragments *in vitro* indicated below (bar graph) (12). The preferred nucleosome and ORC occupancy distributions are inverse of each other. Regions of GC rich DNA (blue line) and conserved, bent, poly A:T DNA (red box) are indicated (94,124). The red box above indicates the region of greatest hyperacetylation on histones H3 and H4 (H3Ac / H4Ac) that occurs exclusively during stages 10–11 when ORC is bound and the origin is active (70). This acetylated nucleosome resides between the sites of high ORC occupancy, with one corresponding to Ori-β, the preferred replication initiation zone (black dotted line) (89,90). This region lies at the center of a diminishing gradient of ORC occupancy and histone acetylation that extends outwards over ∼20 kb epigenome domain (red arrows below) (66,70). These data lead to a model wherein both DNA sequence and nucleosome acetylation contribute to the location and developmental timing of pre-RC assembly at the amplicons.

Further analysis of nucleotide composition showed that the nucleosome occupied regions were relatively G:C rich while the NDRs were very A:T rich (Figure 6B and C). The NDRs in ACE3 and Ori-β include extended poly A:T tracts that are highly conserved at orthologous amplicons in the genus *Drosophila* (92–94). Although these poly A:T regions in the NDRs of ACE3 and Ori-β are strongly disfavored by nucleosomes, previous evidence suggested that they are bound by ORC *in vitro* and *in vivo* (12–13,18,66,68,70,109). To further examine the DNA sequence contribution to nucleosome and ORC positions, we compared our MNase-Seq data for nucleosome position *in vivo* to ORC binding to DAFC-66D DNA *in vitro* from the Botchan lab (12). Strikingly, the quantitative profile of ORC binding to different naked DNA fragments across DAFC-66D *in vitro* is the inverse of the observed nucleosome positions *in vivo* (Figure 7). The poly A:T rich regions in ACE3 and Ori-β are preferred by ORC but strongly disfavored by nucleosomes (Figure 7). These results are consistent with the idea that nucleosomes and ORC prefer different DNA sequences, and that this contributes to their positions at DAFC-66D and perhaps other origins.

## DISCUSSION

We have defined nucleosome position at amplicon origins to evaluate how chromatin influences origin activity during development. At the well-characterized DAFC-66D origin, ORC binding sites and other sequences important for origin function are depleted of nucleosomes. Our data from tissues are consistent with evidence from cells in culture that ORC binds in NDRs, but also confirms that being an NDR is not sufficient for ORC binding and origin activity. Importantly, integration of this data with previous findings suggests that DNA sequence and structure contributes to ORC and nucleosome positions at DAFC-66D. Hyperacetylation of these positioned nucleosomes during stage 10 of oogenesis likely promotes ORC recruitment and pre-RC assembly onto adjacent DNA sequences that are disfavored by nucleosomes but preferred by ORC. More broadly, these data at DAFC-66D suggest that at a subclass of origins DNA sequence and structure in conjunction with nucleosome modification promote pre-RC assembly.

### DNA sequences essential for amplicon origin function are NDRs, but NDRs are not sufficient to specify the origin

Similar to other origins from yeast to humans, ORC binding sites at DAFC-66D are NDRs (Figure 7) (2). The extended depletion of nucleosomes in Ori-β also likely permits the clamping of the MCM replicative helicase around the DNA at this preferred replication initiation site. Thus, although amplicon origins are responsible for a specific developmental gene amplification, they share many attributes with other origins including nucleosome depletion at pre-RC binding sites. Given that ORC has relaxed DNA binding specificity *in vitro*, an extreme view of origin specification posits that ORC can bind DNA wherever it is not occluded by nucleosomes. Our data, however, are not consistent with this extreme ‘permissive’ model. Although NDRs at the amplicons are similar among all stages of oogenesis and in S2 cells, ORC is only bound to the amplicons during stages 10–11 of oogenesis. It remains possible that other proteins bound to the amplicon NDRs prevent ORC access to DNA when the origin is not active. Nonetheless, genome-wide analysis of nucleosome position and ORC binding indicated that while ORC binding sites are NDRs, not all NDRs are bound by ORC, consistent with the view that depletion of nucleosomes is not sufficient to specify ORC binding sites (36,110).

Evidence suggests that ATP-dependent nucleosome remodeler complexes may regulate some origins of eukaryotic chromosomes and mammalian viruses (37,111–114). We found no evidence, however, that a change in nucleosome position is associated with the developmental activation of amplicon origins. It remains an open question whether small changes to nucleosome position, or a change in the dynamic association of nucleosomes with origin DNA, contribute to the developmental activation of the origins. Indeed, it has been shown that nucleosomes that contain both histone variants H3.3 and H2Az have a labile association with DNA and correlate with ORC binding sites genome wide (36,115–117). Given that these double-variant nucleosomes are not detected under standard conditions for MNase-Seq, other approaches will be needed to determine whether they are resident at amplicon origins and contribute to their activity (118,119).

### Origin DNA sequence and structure may contribute to adjacent nucleosome and ORC positions

Our analysis leads us to propose that DNA primary sequence contributes to a close apposition of nucleosomes and ORC at DAFC-66D. We found that the DNA code for nucleosome positioning closely matches our observed nucleosome occupancy *in vivo* (104,105). The NDRs at ACE3 and Ori-β both contain poly A:T tracts, a DNA sequence type that is strongly disfavored by nucleosomes, and which often resides at the borders between nucleosome-occupied and unoccupied regions (120). The two poly A:T rich regions at DAFC-66D flank the nucleosome-preferred GC-rich sequences, which all together likely contribute to the observed positioning of nucleosomes over the cp18 gene. Conversely, it has been shown that yeast, human, and *Drosophila* ORC prefers to bind to poly A:T DNA, and that ORC has higher affinity for the AT-rich ACE3 and Ori-β than the GC-rich cp18 transcription unit *in vitro* (12,18,68,109,121). The inverse relationship between ORC binding *in vitro* and nucleosome positions *in vivo* suggests that ORC binds these NDRs not just because they lack nucleosomes, but also because ORC favors the DNA sequences that are disfavored by nucleosomes (Figure 7) (12). An important contribution of the poly A:T sequences to origin function is supported by the ability of DAFC-66D transgenes that contain them to direct amplification at ectopic genomic sites, and by their high level of conservation at orthologous amplicons in other *Drosophila* species that diverged 40 million years ago (90,92–94,107). One prediction from our data is that these conserved poly A:T sequences template similar nucleosome and ORC positions in these distant *Drosophila* species. Moreover, our data for DNA sequence contributing to nucleosome and ORC positions may be more broadly relevant to reports of specific arrangements of GC and AT-rich sequences at origins in humans, mice, and flies (17,19,21,122–123).

DNA primary sequence may influence nucleosome and ORC positions in part through an effect on DNA helix topology and higher-order structure (104). It is known that poly A:T tracts form specific conformations that are strongly disfavored for wrapping around nucleosomes, and that poly A:T stretches at DAFC-66D adopt this type of DNA structure *in vitro* (120,124). In addition, it was previously shown that *Drosophila* ORC prefers to bind negatively supercoiled DNA (12). While an intrinsic DNA bending and supercoiling is disfavored by nucleosomes, it may promote a specific path of the DNA fiber through a central channel of the ORC, a proposed conformation that is consistent with EM and AFM imaging and the recent crystal structure of the *Drosophila* ORC (125–129). Indeed, other evidence from both eukaryotes and prokaryotes suggests that DNA bending may be an ancient origin property that is important for origin function (2,130–139). Importantly, our results suggest the new idea that this intrinsic DNA architecture at DAFC-66D may help choreograph the interplay between ORC and origin nucleosomes.

### Hyperacetylation of positioned nucleosomes may establish an amplicon origin epigenome domain that stimulates pre-RC assembly and activation

Although ORC prefers to bind ACE3 and Ori-β, this preference is only ∼7-fold relative to other sequences, with negative supercoiling of DNA increasing ORC binding specificity to ∼30-fold (12). Given that these sequences are NDRs before stages 10–11, this intrinsic preference of ORC for DNA sequence, and perhaps DNA structure, is not sufficient to explain the developmental specificity of the amplicon origins. Previous data indicated that histone hyperacetylation during stages 10–11 is required for ORC binding and amplicon activity (66,70–73). Recent ChIP-qPCR and ChIP-array analysis revealed that the highest histone hyperacetylation and ORC occupancy occurs near the preferred initiation sites at all amplicon loci, but that there is also a gradient of decreasing histone acetylation and ORC binding that extends outward over ∼10–20 kb (Figure 7) (66,70). At DAFC-66D, a highly prominent peak of hyperacetylation corresponds to the nucleosomes that are positioned over cp18, in between ACE3 and Ori-β, the two sites of highest ORC occupancy (Figure 7) (70). These results lead us to propose that hyperacetylation of these positioned nucleosomes during stage 10 may promote ORC recruitment and pre-RC assembly onto the adjacent NDRs of ACE3 and Ori-β. These initial events at the center of the amplicon may nucleate the observed 20 kb epigenome domain of histone acetylation and ORC binding to intergenic NDRs, which may adopt a higher order structure that promotes origin activity (2,66,70). Important remaining questions include how HATs are recruited to the amplicons and how histone acetylation facilitates different steps of pre-RC assembly and activation. The developmental timing of histone hyperacetylation is correlated with pre-RC assembly at active amplicons in other *Drosophila* species, further suggesting that this conserved aspect of the origin epigenome is important for origin function (94).

### DAFC-66D may belong to a subclass of origins at which nucleosomes promote pre-RC assembly

Our data for DAFC-66D is consistent with growing evidence that nucleosomes can play a direct, positive role in pre-RC assembly at a subclass of origins from yeast to humans (46,49–51,140–143). We propose that at these other origins DNA sequence templates adjacent nucleosome and ORC positions. Indeed, a correlation between poly A:T tracts and NDRs at replication origins has been noted in yeast, humans and *Drosophila* (36,43,45). While this paper was under review, a high-resolution mapping of DNA replication initiation sites was reported for three *Drosophila* cell culture lines (144). Consistent with our data, analysis of these origins in cell culture led to the conclusion that poly A:T-rich sequences, DNA shape, and chromatin modifications all contribute to specifying origins. An important prediction from our results is that these poly A:T sequences not only define NDR boundaries, but also promote the binding of ORC adjacent to some nucleosomes that directly assist pre-RC assembly. ORC may have evolved a relaxed preference for DNA sequences that are disfavored by nucleosomes because of selective pressure to assemble an excess of pre-RCs to ensure full genome duplication. Given that even a relatively short poly A:T sequence promotes nucleosome exclusion over an ∼100–150 bp region, the preference of ORC for these sequences may also ensure an NDR of sufficient size for the clamping of the MCM helicase around DNA (4,145). Our high-resolution MNase-Seq maps, combined with other methods afforded by the model amplicon origins, will permit a further definition of how nucleosome position and modification promotes pre-RC assembly at a subclass of origins.

## Supplementary Material

SUPPLEMENTARY DATA

## ACCESSION NUMBER

NCBI-SRA BioProject SRP057811.

## References

[1] Hills S.A., Diffley J.F. (2014). DNA replication and oncogene-induced replicative stress. Curr. Biol..

[2] Mechali M., Yoshida K., Coulombe P., Pasero P. (2013). Genetic and epigenetic determinants of DNA replication origins, position and activation. Curr. Opin. Genet. Dev..

[3] Remus D., Diffley J.F. (2009). Eukaryotic DNA replication control: lock and load, then fire. Curr. Opin. Cell Biol..

[4] Bell S.D., Botchan M.R. (2013). The minichromosome maintenance replicative helicase. Cold Spring Harbor Perspect. Biol..

[5] Frigola J., Remus D., Mehanna A., Diffley J.F. (2013). ATPase-dependent quality control of DNA replication origin licensing. Nature.

[6] Samson R.Y., Bell S.D. (2013). MCM loading—an open-and-shut case. Mol Cell.

[7] Coster G., Frigola J., Beuron F., Morris E.P., Diffley J.F. (2014). Origin licensing requires ATP binding and hydrolysis by the MCM replicative helicase. Mol. Cell.

[8] On K.F., Beuron F., Frith D., Snijders A.P., Morris E.P., Diffley J.F. (2014). Prereplicative complexes assembled in vitro support origin-dependent and independent DNA replication. EMBO J..

[9] Samel S.A., Fernandez-Cid A., Sun J., Riera A., Tognetti S., Herrera M.C., Li H., Speck C. (2014). A unique DNA entry gate serves for regulated loading of the eukaryotic replicative helicase MCM2–7 onto DNA. Genes Dev..

[10] Yeeles J.T., Deegan T.D., Janska A., Early A., Diffley J.F. (2015). Regulated eukaryotic DNA replication origin firing with purified proteins. Nature.

[11] Weinreich M. (2015). Molecular biology: DNA replication reconstructed. Nature.

[12] Remus D., Beall E.L., Botchan M.R. (2004). DNA topology, not DNA sequence, is a critical determinant for Drosophila ORC-DNA binding. EMBO J..

[13] Bielinsky A.-K., Blitzblau H., Beall E.L., Ezrokhi M., Smith H.S., Botchan M.R., Gerbi S.A. (2001). Origin recognition complex binding to a metazoan replication origin. Curr. Biol..

[14] Chesnokov I., Remus D., Botchan M. (2001). Functional analysis of mutant and wild-type Drosophila origin recognition complex. Proc. Natl. Acad. Sci. U.S.A..

[15] Vashee S., Cvetic C., Lu W., Simancek P., Kelly T.J., Walter J.C. (2003). Sequence-independent DNA binding and replication initiation by the human origin recognition complex. Genes Dev..

[16] Aladjem M.I. (2007). Replication in context: dynamic regulation of DNA replication patterns in metazoans. Nat. Rev. Genet..

[17] Cayrou C., Coulombe P., Vigneron A., Stanojcic S., Ganier O., Peiffer I., Rivals E., Puy A., Laurent-Chabalier S., Desprat R. (2011). Genome-scale analysis of metazoan replication origins reveals their organization in specific but flexible sites defined by conserved features. Genome Res..

[18] Balasov M., Huijbregts R.P., Chesnokov I. (2007). Role of the Orc6 protein in origin recognition complex-dependent DNA binding and replication in Drosophila melanogaster. Mol. Cell. Biol..

[19] Besnard E., Babled A., Lapasset L., Milhavet O., Parrinello H., Dantec C., Marin J.M., Lemaitre J.M. (2012). Unraveling cell type-specific and reprogrammable human replication origin signatures associated with G-quadruplex consensus motifs. Nat. Struct. Mol. Biol..

[20] Gilbert D.M. (2012). Replication origins run (ultra) deep. Nat. Struct. Mol. Biol..

[21] Cayrou C., Coulombe P., Puy A., Rialle S., Kaplan N., Segal E., Mechali M. (2012). New insights into replication origin characteristics in metazoans. Cell Cycle.

[22] Edenberg H.J., Huberman J.A. (1975). Eukaryotic chromosome replication. Annu. Rev. Genet..

[23] Nordman J., Orr-Weaver T.L. (2012). Regulation of DNA replication during development. Development.

[24] Mechali M. (2010). Eukaryotic DNA replication origins: many choices for appropriate answers. Nat. Rev. Mol. Cell Biol..

[25] Hyrien O., Maric C., Mechali M. (1995). Transition in specification of embryonic metazoan DNA replication origins. Science.

[26] Mechali M., Kearsey S. (1984). Lack of specific sequence requirement for DNA replication in Xenopus eggs compared with high sequence specificity in yeast. Cell.

[27] Aladjem M.I., Falaschi A., Kowalski D., Depamphilis ML (2006). DNA Replication and Human Disease.

[28] Dai J., Chuang R.Y., Kelly T.J. (2005). DNA replication origins in the Schizosaccharomyces pombe genome. Proc. Natl. Acad. Sci. U.S.A..

[29] Huberman J.A. (1968). Visualization of replicating mammalian and T4 bacteriophage DNA. Cold Spring Harbor Symp. Quant. Biol..

[30] Lima-de-Faria A., Jaworska H. (1968). Late DNA synthesis in heterochromatin. Nature.

[31] Hiratani I., Ryba T., Itoh M., Rathjen J., Kulik M., Papp B., Fussner E., Bazett-Jones D.P., Plath K., Dalton S. (2010). Genome-wide dynamics of replication timing revealed by in vitro models of mouse embryogenesis. Genome Res..

[32] Schwaiger M., Kohler H., Oakeley E.J., Stadler M.B., Schubeler D. (2010). Heterochromatin protein 1 (HP1) modulates replication timing of the Drosophila genome. Genome Res..

[33] Bell O., Schwaiger M., Oakeley E.J., Lienert F., Beisel C., Stadler M.B., Schubeler D. (2010). Accessibility of the Drosophila genome discriminates PcG repression, H4K16 acetylation and replication timing. Nat. Struct. Mol. Biol..

[34] Schwaiger M., Stadler M.B., Bell O., Kohler H., Oakeley E.J., Schubeler D. (2009). Chromatin state marks cell-type- and gender-specific replication of the Drosophila genome. Genes Dev..

[35] Hiratani I., Ryba T., Itoh M., Yokochi T., Schwaiger M., Chang C.W., Lyou Y., Townes T.M., Schubeler D., Gilbert D.M. (2008). Global reorganization of replication domains during embryonic stem cell differentiation. PLoS Biol..

[36] MacAlpine H.K., Gordan R., Powell S.K., Hartemink A.J., MacAlpine D.M. (2010). Drosophila ORC localizes to open chromatin and marks sites of cohesin complex loading. Genome Res..

[37] Eaton M.L., Prinz J.A., MacAlpine H.K., Tretyakov G., Kharchenko P.V., MacAlpine D.M. (2011). Chromatin signatures of the Drosophila replication program. Genome Res..

[38] Cadoret J.C. (2008). Genome-wide studies highlight indirect links between human replication origins and gene regulation. Proc. Natl. Acad. Sci. U.S.A..

[39] Kim S.M., Dubey D.D., Huberman J.A. (2003). Early-replicating heterochromatin. Genes Dev..

[40] Gilbert D.M. (2010). Evaluating genome-scale approaches to eukaryotic DNA replication. Nat. Rev. Genet..

[41] Hayashi M.T., Takahashi T.S., Nakagawa T., Nakayama J., Masukata H. (2009). The heterochromatin protein Swi6/HP1 activates replication origins at the pericentromeric region and silent mating-type locus. Nat. Cell Biol..

[42] Lubelsky Y., Sasaki T., Kuipers M.A., Lucas I., Le Beau M.M., Carignon S., Debatisse M., Prinz J.A., Dennis J.H., Gilbert D.M. (2011). Pre-replication complex proteins assemble at regions of low nucleosome occupancy within the Chinese hamster dihydrofolate reductase initiation zone. Nucleic Acids Res..

[43] Berbenetz N.M., Nislow C., Brown G.W. (2010). Diversity of eukaryotic DNA replication origins revealed by genome-wide analysis of chromatin structure. PLoS Genet..

[44] Eaton M.L., Galani K., Kang S., Bell S.P., MacAlpine D.M. (2010). Conserved nucleosome positioning defines replication origins. Genes Dev..

[45] Field Y., Kaplan N., Fondufe-Mittendorf Y., Moore I.K., Sharon E., Lubling Y., Widom J., Segal E. (2008). Distinct modes of regulation by chromatin encoded through nucleosome positioning signals. PLoS Comput. Biol..

[46] Lipford J.R., Bell S.P. (2001). Nucleosomes positioned by ORC facilitate the initiation of DNA replication. Mol. Cell.

[47] Yin S., Deng W., Hu L., Kong X. (2009). The impact of nucleosome positioning on the organization of replication origins in eukaryotes. Biochem. Biophysi. Res. Commun..

[48] Simpson R.T. (1990). Nucleosome positioning can affect the function of a cis-acting DNA element in vivo. Nature.

[49] Hoggard T., Shor E., Muller C.A., Nieduszynski C.A., Fox C.A. (2013). A Link between ORC-origin binding mechanisms and origin activation time revealed in budding yeast. PLoS Genet..

[50] Muller P., Park S., Shor E., Huebert D.J., Warren C.L., Ansari A.Z., Weinreich M., Eaton M.L., MacAlpine D.M., Fox C.A. (2010). The conserved bromo-adjacent homology domain of yeast Orc1 functions in the selection of DNA replication origins within chromatin. Genes Dev..

[51] Kuo A.J., Song J., Cheung P., Ishibe-Murakami S., Yamazoe S., Chen J.K., Patel D.J., Gozani O. (2012). The BAH domain of ORC1 links H4K20me2 to DNA replication licensing and Meier-Gorlin syndrome. Nature.

[52] Tardat M., Brustel J., Kirsh O., Lefevbre C., Callanan M., Sardet C., Julien E. (2010). The histone H4 Lys 20 methyltransferase PR-Set7 regulates replication origins in mammalian cells. Nat. Cell Biol..

[53] Iizuka M., Stillman B. (1999). Histone acetyltransferase HBO1 interacts with the ORC1 subunit of the human initiator protein. J. Biol. Chem..

[54] Burke T.W., Cook J.G., Asano M., Nevins J.R. (2001). Replication factors MCM2 and ORC1 interact with the histone acetyltransferase HBO1. J. Biol. Chem..

[55] Stedman W., Deng Z., Lu F., Lieberman P.M. (2004). ORC, MCM, and histone hyperacetylation at the Kaposi's sarcoma-associated herpesvirus latent replication origin. J. Virol..

[56] Iizuka M., Matsui T., Takisawa H., Smith M.M. (2006). Regulation of replication licensing by acetyltransferase Hbo1. Mol. Cell. Biol..

[57] Iizuka M., Sarmento O.F., Sekiya T., Scrable H., Allis C.D., Smith M.M. (2008). Hbo1 Links p53-dependent stress signaling to DNA replication licensing. Mol. Cell. Biol..

[58] Miotto B., Struhl K. (2008). HBO1 histone acetylase is a coactivator of the replication licensing factor Cdt1. Genes Dev..

[59] Wu Z.Q., Liu X. (2008). Role for Plk1 phosphorylation of Hbo1 in regulation of replication licensing. Proc. Natl. Acad. Sci. U.S.A..

[60] Iizuka M., Takahashi Y., Mizzen C.A., Cook R.G., Fujita M., Allis C.D., Frierson H.F. Jr, Fukusato T., Smith M.M. (2009). Histone acetyltransferase Hbo1: catalytic activity, cellular abundance, and links to primary cancers. Gene.

[61] Miotto B., Struhl K. (2010). HBO1 histone acetylase activity is essential for DNA replication licensing and inhibited by Geminin. Mol. Cell.

[62] Mantiero D., Mackenzie A., Donaldson A., Zegerman P. (2011). Limiting replication initiation factors execute the temporal programme of origin firing in budding yeast. EMBO J..

[63] Calvi B.R., DePamphilis ML (2006). DNA Replication and Human Disease.

[64] Claycomb J.M., Orr-Weaver T.L. (2005). Developmental gene amplification: insights into DNA replication and gene expression. Trends Genet..

[65] Spradling A. (1981). The organization and amplification of two chromosomal domains containing Drosophila chorion genes. Cell.

[66] Kim J.C., Nordman J., Xie F., Kashevsky H., Eng T., Li S., MacAlpine D.M., Orr-Weaver T.L. (2011). Integrative analysis of gene amplification in Drosophila follicle cells: parameters of origin activation and repression. Genes Dev..

[67] Calvi B.R., Lilly M.A., Spradling A.C. (1998). Cell cycle control of chorion gene amplification. Genes Dev..

[68] Austin R.J., Orr-Weaver T.L., Bell S.P. (1999). Drosophila ORC specifically binds to ACE3, an origin of DNA replication control element. Genes Dev..

[69] Royzman I., Austin R.J., Bosco G., Bell S.P., Orr-Weaver T.L. (1999). ORC localization in Drosophila follicle cells and the effects of mutations in *dE2F* and *dDP*. Genes Dev..

[70] Liu J., McConnell K., Dixon M., Calvi B.R. (2012). Analysis of model replication origins in Drosophila reveals new aspects of the chromatin landscape and its relationship to origin activity and the prereplicative complex. Mol. Biol. Cell.

[71] Aggarwal B.D., Calvi B.R. (2004). Chromatin regulates origin activity in Drosophila follicle cells. Nature.

[72] Hartl T., Boswell C., Orr-Weaver T.L., Bosco G. (2007). Developmentally regulated histone modifications in Drosophila follicle cells: initiation of gene amplification is associated with histone H3 and H4 hyperacetylation and H1 phosphorylation. Chromosoma.

[73] McConnell K.H., Dixon M., Calvi B.R. (2012). The histone acetyltransferases CBP and Chameau integrate developmental and DNA replication programs in Drosophila ovarian follicle cells. Development.

[74] Beall E.L. (2002). Role for a Drosophila Myb-containing protein complex in site-specific DNA replication. Nature.

[75] Lewis P.W., Beall E.L., Fleischer T.C., Georlette D., Link A.J., Botchan M.R. (2004). Identification of a Drosophila Myb-E2F2/RBF transcriptional repressor complex. Genes Dev..

[76] de Nooij J., Letendre M., Hariharan I. (1996). A cyclin-dependent kinase inhibitor, Dacapo, is necessary for timely exit from the cell cycle during Drosophila embryogenesis. Cell.

[77] Flores O., Orozco M. (2011). nucleR: a package for non-parametric nucleosome positioning. Bioinformatics.

[78] Roy S., Ernst J., Kharchenko P.V., Kheradpour P., Negre N., Eaton M.L., Landolin J.M., Bristow C.A., Ma L., Lin M.F. (2010). Identification of functional elements and regulatory circuits by Drosophila modENCODE. Science.

[79] Gilchrist D.A., Dos Santos G., Fargo D.C., Xie B., Gao Y., Li L., Adelman K. (2010). Pausing of RNA polymerase II disrupts DNA-specified nucleosome organization to enable precise gene regulation. Cell.

[80] Rastogi P.A. (2000). MacVector. Integrated sequence analysis for the Macintosh. Methods Mol. Biol..

[81] Negre N., Brown C.D., Ma L., Bristow C.A., Miller S.W., Wagner U., Kheradpour P., Eaton M.L., Loriaux P., Sealfon R. (2011). A cis-regulatory map of the Drosophila genome. Nature.

[82] Henikoff J.G., Belsky J.A., Krassovsky K., MacAlpine D.M., Henikoff S. (2011). Epigenome characterization at single base-pair resolution. Proc. Natl. Acad. Sci. U.S.A..

[83] Floer M., Wang X., Prabhu V., Berrozpe G., Narayan S., Spagna D., Alvarez D., Kendall J., Krasnitz A., Stepansky A. (2010). A RSC/nucleosome complex determines chromatin architecture and facilitates activator binding. Cell.

[84] Tims H.S., Gurunathan K., Levitus M., Widom J. (2011). Dynamics of nucleosome invasion by DNA binding proteins. J. Mol. Biol..

[85] Drysdale R., FlyBase C. (2008). FlyBase : a database for the Drosophila research community. Methods Mol. Biol..

[86] Dos Santos G., Schroeder A.J., Goodman J.L., Strelets V.B., Crosby M.A., Thurmond J., Emmert D.B., Gelbart W.M., FlyBase C. (2015). FlyBase: introduction of the Drosophila melanogaster Release 6 reference genome assembly and large-scale migration of genome annotations. Nucleic Acids Res..

[87] Delidakis C., Kafatos F. (1989). Amplification enhancers and replication origins in the autosomal chorion gene cluster of Drosophila. EMBO J..

[88] Orr-Weaver T., Johnston C., Spradling A. (1989). The role of ACE3 in Drosophila chorion gene amplification. EMBO J..

[89] Heck M., Spradling A. (1990). Multiple replication origins are used during Drosophila chorion gene amplification. J. Cell Biol..

[90] Zhang H., Tower J. (2004). Sequence requirements for function of the Drosophila chorion gene locus ACE3 replicator and ori-beta origin elements. Development.

[91] Swimmer C., Delidakis C., Kafatos F. (1989). Amplification-control element ACE-3 is important but not essential for autosomal chorion gene amplification. Proc. Natl. Acad. Sci. U.S.A..

[92] Fenerjian M., Martinez-Cruzado J., Swimmer C., King D., Kafatos F. (1989). Evolution of the autosomal chorion cluster in Drosophila. II. Chorion gene expression and sequence comparisons of the s16 and s19 genes in evolutionarily distant species. J. Mol. Evol..

[93] Swimmer C., Fenerjian M., Martinez-Cruzado J., Kafatos F. (1990). Evolution of the autosomal chorion cluster in Drosophila. III. Comparison of the s18 gene in evolutionarily distant species and heterospecific control of chorion gene amplification. J. Mol. Biol..

[94] Calvi B.R., Byrnes B.A., Kolpakas A.J. (2007). Conservation of epigenetic regulation, ORC binding and developmental timing of DNA replication origins in the genus Drosophila. Genetics.

[95] Parks S., Wakimoto B., Spradling A. (1986). Replication and expression of an X-linked cluster of Drosophila chorion genes. Dev. Biol..

[96] Spradling A., de C.D., Wakimoto B., Levine J., Kalfayan L., Cooley L. (1987). Amplification of the X-linked Drosophila chorion gene cluster requires a region upstream from the s38 chorion gene. EMBO J..

[97] Lane M., Sauer K., Wallace K., Jan Y., Lehner C., Vaessin H. (1996). Dacapo, a cyclin-dependent kinase inhibitor, stops cell proliferation during Drosophila development. Cell.

[98] Claycomb J.M., Benasutti M., Bosco G., Fenger D.D., Orr-Weaver T.L. (2004). Gene amplification as a developmental strategy: isolation of two developmental amplicons in Drosophila. Dev. Cell.

[99] Hua B.L., Li S., Orr-Weaver T.L. (2014). The role of transcription in the activation of a Drosophila amplification origin. G3.

[100] Kim J.C., Orr-Weaver T.L. (2011). Analysis of a Drosophila amplicon in follicle cells highlights the diversity of metazoan replication origins. Proc. Natl. Acad. Sci. U.S.A..

[101] Xie F., Orr-Weaver T.L. (2008). Isolation of a Drosophila amplification origin developmentally activated by transcription. Proc. Natl. Acad. Sci. U.S.A..

[102] Clapier C.R., Cairns B.R. (2009). The biology of chromatin remodeling complexes. Annu. Rev. Biochem..

[103] Macalpine D.M., Rodriguez H.K., Bell S.P. (2004). Coordination of replication and transcription along a Drosophila chromosome. Genes Dev..

[104] Segal E., Fondufe-Mittendorf Y., Chen L., Thastrom A., Field Y., Moore I.K., Wang J.P., Widom J. (2006). A genomic code for nucleosome positioning. Nature.

[105] Kaplan N., Moore I.K., Fondufe-Mittendorf Y., Gossett A.J., Tillo D., Field Y., LeProust E.M., Hughes T.R., Lieb J.D., Widom J. (2009). The DNA-encoded nucleosome organization of a eukaryotic genome. Nature.

[106] Valouev A., Johnson S.M., Boyd S.D., Smith C.L., Fire A.Z., Sidow A. (2011). Determinants of nucleosome organization in primary human cells. Nature.

[107] de Cicco D., Spradling A. (1984). Localization of a cis-acting element responsible for the developmentally regulated amplification of Drosophila chorion genes. Cell.

[108] Lu L., Tower J. (1997). A transcriptional insulator element, the su(Hw) binding site, protects a chromosomal DNA replication origin form position effects. Mol. Cell. Biol..

[109] Liu S., Balasov M., Wang H., Wu L., Chesnokov I.N., Liu Y. (2011). Structural analysis of human Orc6 protein reveals a homology with transcription factor TFIIB. Proc. Natl. Acad. Sci. U.S.A..

[110] Lubelsky Y., Prinz J.A., DeNapoli L., Li Y., Belsky J.A., MacAlpine D.M. (2014). DNA replication and transcription programs respond to the same chromatin cues. Genome Res..

[111] Flanagan J.F., Peterson C.L. (1999). A role for the yeast SWI/SNF complex in DNA replication. Nucleic Acids Res..

[112] Alexiadis V., Varga-Weisz P.D., Bonte E., Becker P.B., Gruss C. (1998). In vitro chromatin remodelling by chromatin accessibility complex (CHRAC) at the SV40 origin of DNA replication. EMBO J..

[113] Avolio-Hunter T.M., Lewis P.N., Frappier L. (2001). Epstein-Barr nuclear antigen 1 binds and destabilizes nucleosomes at the viral origin of latent DNA replication. Nucleic Acids Res..

[114] Sugimoto N., Yugawa T., Iizuka M., Kiyono T., Fujita M. (2011). Chromatin remodeler sucrose nonfermenting 2 homolog (SNF2H) is recruited onto DNA replication origins through interaction with Cdc10 protein-dependent transcript 1 (Cdt1) and promotes pre-replication complex formation. J. Biol. Chem..

[115] Mito Y., Henikoff J.G., Henikoff S. (2005). Genome-scale profiling of histone H3.3 replacement patterns. Nat. Genet..

[116] Deal R.B., Henikoff J.G., Henikoff S. (2010). Genome-wide kinetics of nucleosome turnover determined by metabolic labeling of histones. Science.

[117] Jin C., Felsenfeld G. (2007). Nucleosome stability mediated by histone variants H3.3 and H2A. Z. Genes Dev..

[118] Henikoff S. (2009). Labile H3.3+H2A.Z nucleosomes mark ‘nucleosome-free regions’. Nat. Genet..

[119] Jin C., Zang C., Wei G., Cui K., Peng W., Zhao K., Felsenfeld G. (2009). H3.3/H2A.Z double variant-containing nucleosomes mark ‘nucleosome-free regions’ of active promoters and other regulatory regions. Nat. Genet..

[120] Segal E., Widom J. (2009). Poly(dA:dT) tracts: major determinants of nucleosome organization. Curr. Opin. Struct. Biol..

[121] Vashee S. (2003). Sequence-independent DNA binding and replication initiation by the human origin recognition complex. Genes Dev..

[122] Rein T., Zorbas H., DePamphilis M.L. (1997). Active mammalian replication origins are associated with a high-density cluster of mCpG dinucleotides. Mol. Cell. Biol.

[123] Antequera F., Bird A. (1999). CpG islands as genomic footprints of promoters that are associated with replication origins. Curr. Biol..

[124] Gimenes F., Assis M.A., Fiorini A., Mareze V.A., Monesi N., Fernandez M.A. (2009). Intrinsically bent DNA sites in the Drosophila melanogaster third chromosome amplified domain. Mol. Genet. Genomics: MGG.

[125] Gaczynska M., Osmulski P.A., Jiang Y., Lee J.K., Bermudez V., Hurwitz J. (2004). Atomic force microscopic analysis of the binding of the Schizosaccharomyces pombe origin recognition complex and the spOrc4 protein with origin DNA. Proc. Natl. Acad. Sci. U.S.A..

[126] Clarey M.G., Botchan M., Nogales E. (2008). Single particle EM studies of the Drosophila melanogaster origin recognition complex and evidence for DNA wrapping. J. Struct. Biol..

[127] Sun J., Kawakami H., Zech J., Speck C., Stillman B., Li H. (2012). Cdc6-induced conformational changes in ORC bound to origin DNA revealed by cryo-electron microscopy. Structure.

[128] Sun J., Evrin C., Samel S.A., Fernandez-Cid A., Riera A., Kawakami H., Stillman B., Speck C., Li H. (2013). Cryo-EM structure of a helicase loading intermediate containing ORC-Cdc6-Cdt1-MCM2–7 bound to DNA. Nat. Struct. Mol. Biol..

[129] Bleichert F., Botchan M.R., Berger J.M. (2015). Crystal structure of the eukaryotic origin recognition complex. Nature.

[130] Dueber E.C., Costa A., Corn J.E., Bell S.D., Berger J.M. (2011). Molecular determinants of origin discrimination by Orc1 initiators in archaea. Nucleic Acids Res..

[131] Dueber E.L., Corn J.E., Bell S.D., Berger J.M. (2007). Replication origin recognition and deformation by a heterodimeric archaeal Orc1 complex. Science.

[132] Marilley M. (2000). Structure-function relationships in replication origins of the yeast Saccharomyces cerevisiae: higher-order structural organization of DNA in regions flanking the ARS consensus sequence. Mol. Gen. Genet.: MGG.

[133] Snyder M., Buchman A.R., Davis R.W. (1986). Bent DNA at a yeast autonomously replicating sequence. Nature.

[134] Hertz G.Z., Young M.R., Mertz J.E. (1987). The A+T-rich sequence of the simian virus 40 origin is essential for replication and is involved in bending of the viral DNA. J. Virol..

[135] Lu Y., Weers B.D., Stellwagen N.C. (2003). Analysis of the intrinsic bend in the M13 origin of replication by atomic force microscopy. Biophys. J..

[136] Zahn K., Blattner F.R. (1987). Direct evidence for DNA bending at the lambda replication origin. Science.

[137] Ohyama T., Nagumo M., Sakuma S. (1989). Study on the function of the bent DNA within adenovirus type 2 EIA enhancer: analysis using an in vitro transcription system. Nucleic Acids Symp. Ser..

[138] Caddle M.S., Lussier R.H., Heintz N.H. (1990). Intramolecular DNA triplexes, bent DNA and DNA unwinding elements in the initiation region of an amplified dihydrofolate reductase replicon. J. Mol. Biol..

[139] Altman A.L., Fanning E. (2004). Defined sequence modules and an architectural element cooperate to promote initiation at an ectopic mammalian chromosomal replication origin. Mol. Cell. Biol..

[140] Noguchi K., Vassilev A., Ghosh S., Yates J.L., DePamphilis M.L. (2006). The BAH domain facilitates the ability of human Orc1 protein to activate replication origins in vivo. EMBO J..

[141] Bicknell L.S., Bongers E.M., Leitch A., Brown S., Schoots J., Harley M.E., Aftimos S., Al-Aama J.Y., Bober M., Brown P.A. (2011). Mutations in the pre-replication complex cause Meier-Gorlin syndrome. Nat. Genet..

[142] Bicknell L.S., Walker S., Klingseisen A., Stiff T., Leitch A., Kerzendorfer C., Martin C.A., Yeyati P., Al Sanna N., Bober M. (2011). Mutations in ORC1, encoding the largest subunit of the origin recognition complex, cause microcephalic primordial dwarfism resembling Meier-Gorlin syndrome. Nat. Genet..

[143] Guernsey D.L., Matsuoka M., Jiang H., Evans S., Macgillivray C., Nightingale M., Perry S., Ferguson M., LeBlanc M., Paquette J. (2011). Mutations in origin recognition complex gene ORC4 cause Meier-Gorlin syndrome. Nat. Genet..

[144] Comoglio F., Schlumpf T., Schmid V., Rohs R., Beisel C., Paro R. (2015). High-resolution profiling of Drosophila replication start sites reveals a DNA shape and chromatin signature of metazoan origins. Cell Rep..

[145] Kornberg R.D., Stryer L. (1988). Statistical distributions of nucleosomes: nonrandom locations by a stochastic mechanism. Nucleic Acids Res..

