# Supplementary material for: DNA sequence templates adjacent nucleosome and ORC sites at gene amplification origins in Drosophila
Source: Nucleic Acids Res. 2015 Oct 10;43(18):8746–61. doi: 10.1093/nar/gkv766 (PMC4605296; doi:10.1093/nar/gkv766)
Supplement: SUPPLEMENTARY DATA [file supp_43_18_8746__index.html]

DNA sequence templates adjacent nucleosome and ORC sites at gene amplification origins in Drosophila — DNA sequence templates adjacent nucleosome and ORC sites at gene amplification origins in Drosophila — SUPPLEMENTARY DATA 

# DNA sequence templates adjacent nucleosome and ORC sites at gene amplification origins in *Drosophila*

## SUPPLEMENTARY DATA

- SUPPLEMENTARY DATA
