# Supplementary material for: DNA sequence templates adjacent nucleosome and ORC sites at gene amplification origins in Drosophila
Source: Nucleic Acids Res. 2015 Oct 10;43(18):8746–61. doi: 10.1093/nar/gkv766 (PMC4605296; doi:10.1093/nar/gkv766)
Supplement: SUPPLEMENTARY DATA [file supp_gkv766_nar-01481-x-2015-File009.pdf]

Supplemental Figure Legends

Figure S1. Nucleosome position is similar in biological replicates of wild-type and replication-inhibited stage 10 follicle cells. (A) The distribution of MNase-Seq fragment sizes from Oregon-R stage 10 follicle cells for two biological replicates (OR_s10.1 and OR_s10.2) and follicle cells in which replication was inhibited by *dacapo* expression (dap_s10). Variability in the distribution of paired-end length among replicates is caused by different extents of MNase digestion, but size filtering to enrich for mononucleosome-protected fragments resulting in highly reproducible genome mapping. (B,C) Comparison of nucleosome occupancy at DAFC-66D (B) and DAFC-7F (C) in two OR replicates and *dacapo*-expressing follicle cells.

Figure S2. Nucleosome occupied regions at the minor amplicons in stage 10 follicle cells. (A-D) Nucleosome occupancy during stage 10 is shown for the indicated amplicons. DAFC-22B (A) is not an active origin in the $OR^{modencode}$ strain that we used. Preferred initiation sites (hashed lines) were previously determined by nascent strand mapping or amplified DNA copy number. ORC occupied regions (red boxes) were determined by ORC ChIP-qPCR or ORC ChIP-array (1-3). All amplicons reside within a larger zone of histone acetylation and ORC binding (2,4).

Figure S3. Nucleosome position at the minor amplicons is similar among follicle cells in different stages of oogenesis and in S2 cells. (A-D) Nucleosome occupancy at the indicated amplicons in ovarian follicle cells during stages 1-8, stage 10, stage 12-13, and embryonic S2 cells.

Figure S4. Comparison of NDR sizes at ORC binding sites among different stages and cell types. (A) ORC binding site NDR size distribution as defined by internucleosomal distance (x-axis) versus ORC binding score from modENCODE (y-axis) in follicle cells during stages 1-8 (blue dots) and stage10 (red dots)(5). (B) The size of ORC binding site NDRs in stage 10 (x-axis) versus stages 1-8 (y-axis). Each spot represents an individual ORC NDR. Those on the diagonal are a similar size in stages 10 and 1-8. (C) ORC binding site NDR size distribution (x-axis) versus ORC binding score (y-axis) in S2 cells in culture (blue dots) and follicle cells during

stage 10 (red dots). (D) The size of ORC binding site NDRs in stage 10 (x-axis) versus S2 cells (y-axis).

Supplemental References

1. Xie, F. and Orr-Weaver, T.L. (2008) Isolation of a Drosophila amplification origin developmentally activated by transcription. *Proc Natl Acad Sci U S A*, **105**, 9651-9656.
2. Kim, J.C., Nordman, J., Xie, F., Kashevsky, H., Eng, T., Li, S., MacAlpine, D.M. and Orr-Weaver, T.L. (2011) Integrative analysis of gene amplification in Drosophila follicle cells: parameters of origin activation and repression. *Genes Dev*, **25**, 1384-1398.
3. Kim, J.C. and Orr-Weaver, T.L. (2011) Analysis of a Drosophila amplicon in follicle cells highlights the diversity of metazoan replication origins. *Proc Natl Acad Sci U S A*, **108**, 16681-16686.
4. Liu, J., McConnell, K., Dixon, M. and Calvi, B.R. (2012) Analysis of model replication origins in Drosophila reveals new aspects of the chromatin landscape and its relationship to origin activity and the prereplicative complex. *Molecular biology of the cell*, **23**, 200-212.
5. Roy, S., Ernst, J., Kharchenko, P.V., Kheradpour, P., Negre, N., Eaton, M.L., Landolin, J.M., Bristow, C.A., Ma, L., Lin, M.F. *et al.* (2010) Identification of functional elements and regulatory circuits by Drosophila modENCODE. *Science*, **330**, 1787-1797.

Liu Fig S1

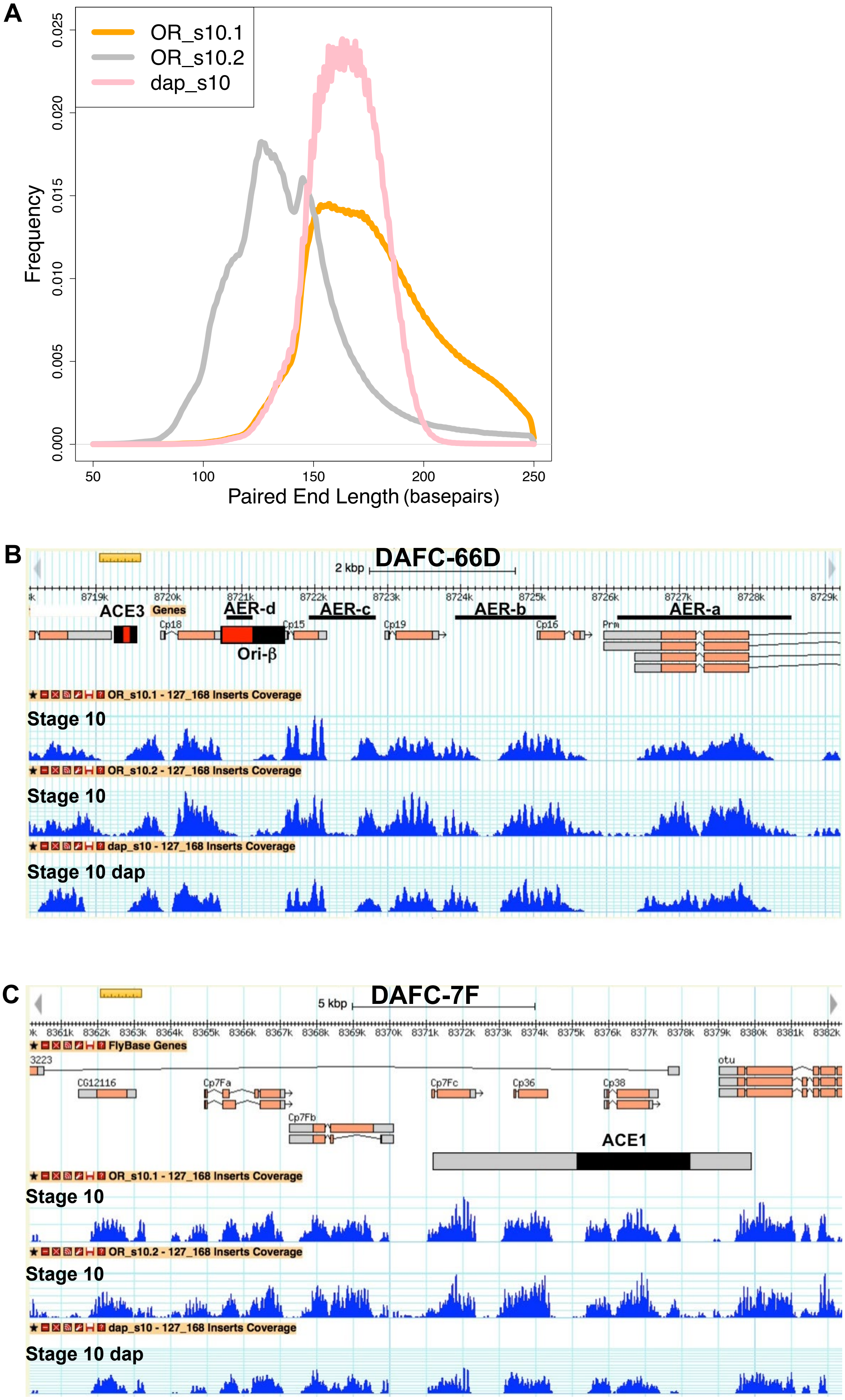
A
OR_s10.1
OR_s10.2
dap_s10
Frequency
0.000
0.005
0.010
0.015
0.020
0.025
50
100
150
200
250
Paired End Length (basepairs)
B
DAFC-66D
2 kbp
ACE3
AER-d
AER-c
AER-b
AER-a
Ori-β
Cp18
Cp15
Cp19
Cp16
OR_s10.1 - 127_168 Inserts Coverage
Stage 10
OR_s10.2 - 127_168 Inserts Coverage
Stage 10
dap_s10 - 127_168 Inserts Coverage
Stage 10 dap
C
DAFC-7F
5 kbp
FlyBase Genes
CG12116
Cp7Fa
Cp7Fb
Cp7Fc
Cp36
Cp38
otu
ACE1
OR_s10.1 - 127_168 Inserts Coverage
Stage 10
OR_s10.2 - 127_168 Inserts Coverage
Stage 10
dap_s10 - 127_168 Inserts Coverage
Stage 10 dap

Liu Fig S2

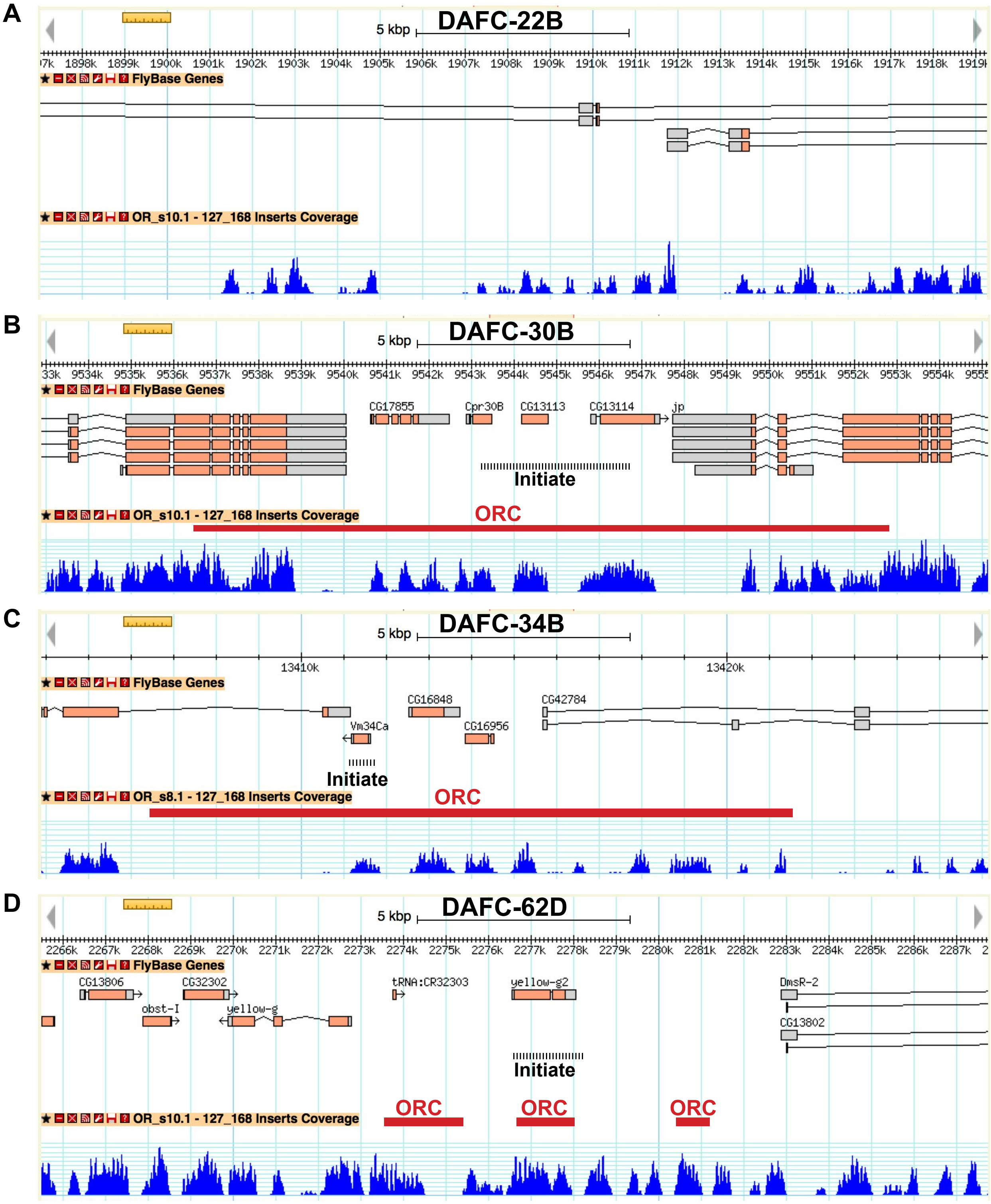

A
DAFC-22B
5 kbp
FlyBase Genes
OR_s10.1 - 127_168 Inserts Coverage
B
DAFC-30B
5 kbp
FlyBase Genes
CG17855
Cpr30B
CG13113
CG13114
jp
Initiate
OR_s10.1 - 127_168 Inserts Coverage
ORC
C
DAFC-34B
5 kbp
13410k
13420k
FlyBase Genes
CG16848
CG42784
Vm34Ca
CG16956
Initiate
OR_s8.1 - 127_168 Inserts Coverage
ORC
D
DAFC-62D
5 kbp
FlyBase Genes
CG13806
CG32302
tRNA:CR32303
yellow-g2
DmsR-2
obst-I
yellow-g
CG13802
Initiate
OR_s10.1 - 127_168 Inserts Coverage
ORC
ORC
ORC

Liu Fig S3

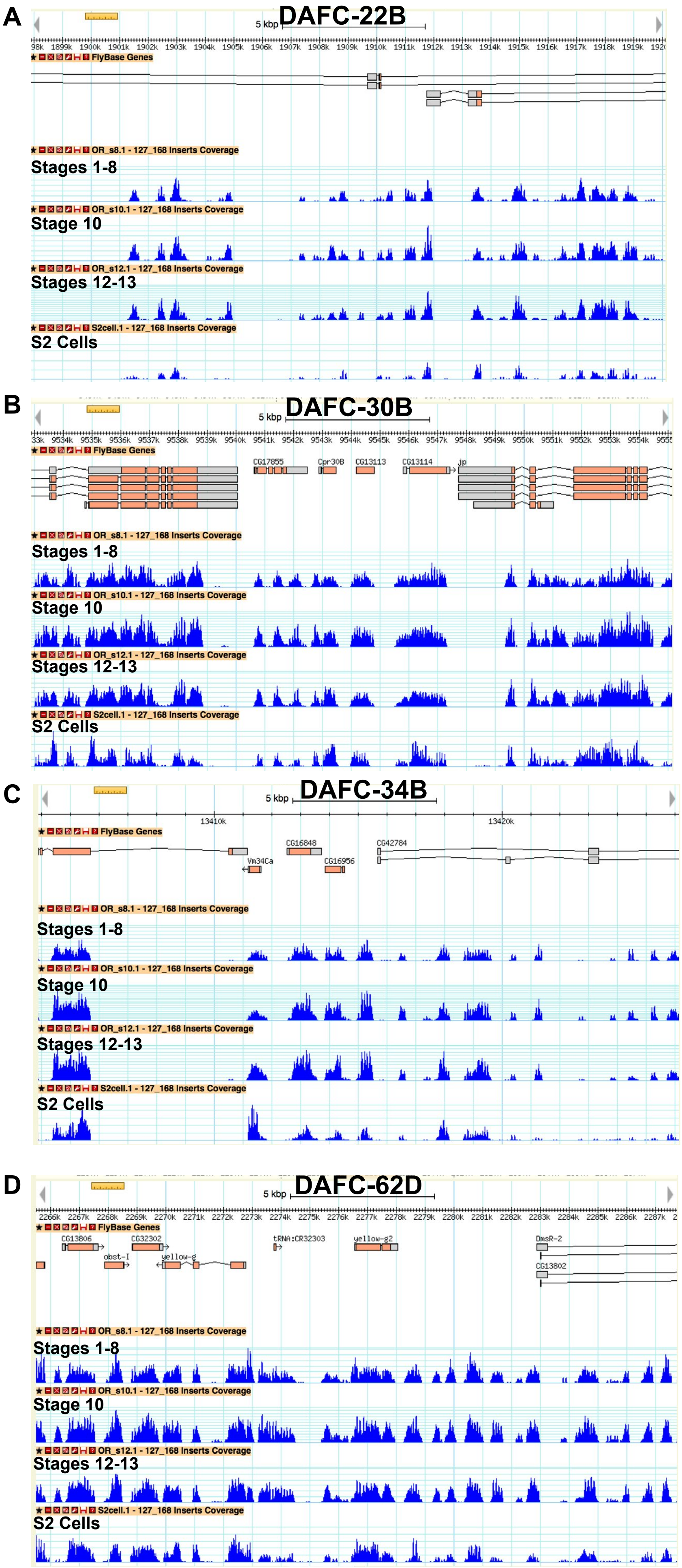
A
DAFC-22B
5 kbp
FlyBase Genes
OR_s8.1 - 127_168 Inserts Coverage
Stages 1-8
OR_s10.1 - 127_168 Inserts Coverage
Stage 10
OR_s12.1 - 127_168 Inserts Coverage
Stages 12-13
S2cell.1 - 127_168 Inserts Coverage
S2 Cells
B
DAFC-30B
5 kbp
FlyBase Genes
CG17855
Cpr30B
CG13113
CG13114
jp
OR_s8.1 - 127_168 Inserts Coverage
Stages 1-8
OR_s10.1 - 127_168 Inserts Coverage
Stage 10
OR_s12.1 - 127_168 Inserts Coverage
Stages 12-13
S2cell.1 - 127_168 Inserts Coverage
S2 Cells
C
DAFC-34B
5 kbp
13410k
13420k
FlyBase Genes
CG16848
CG42784
Vm34Ca
CG16956
OR_s8.1 - 127_168 Inserts Coverage
Stages 1-8
OR_s10.1 - 127_168 Inserts Coverage
Stage 10
OR_s12.1 - 127_168 Inserts Coverage
Stages 12-13
S2cell.1 - 127_168 Inserts Coverage
S2 Cells
D
DAFC-62D
5 kbp
FlyBase Genes
CG13806
CG32302
obst-I
yellow-g
tRNA:CR32303
yellow-g2
DmsR-2
CG13802
OR_s8.1 - 127_168 Inserts Coverage
Stages 1-8
OR_s10.1 - 127_168 Inserts Coverage
Stage 10
OR_s12.1 - 127_168 Inserts Coverage
Stages 12-13
S2cell.1 - 127_168 Inserts Coverage
S2 Cells

Liu Fig S4

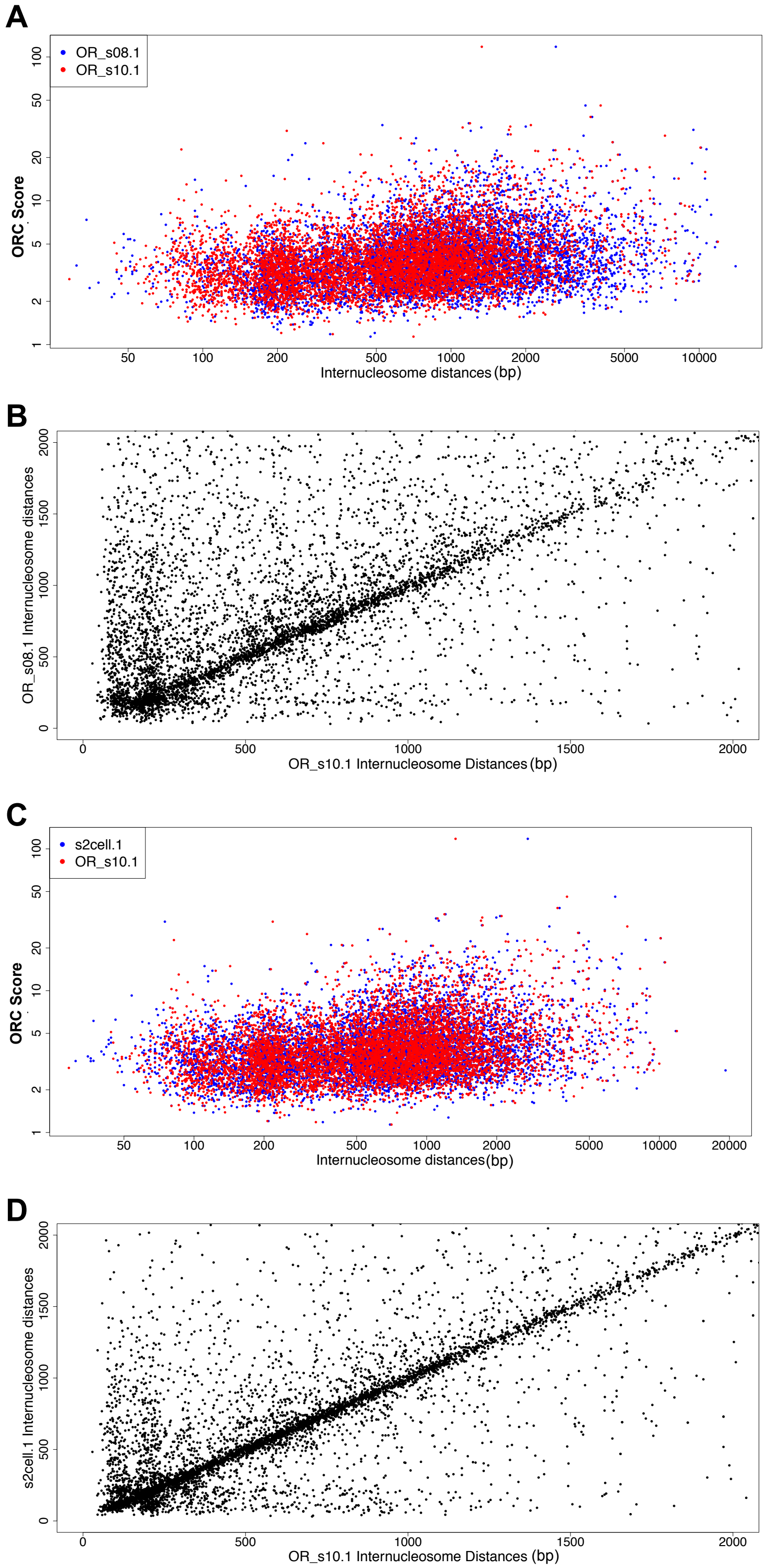
A
OR_s08.1
OR_s10.1
ORC Score
Internucleosome distances (bp)
B
OR_s08.1 Internucleosome distances
OR_s10.1 Internucleosome Distances (bp)
C
s2cell.1
OR_s10.1
ORC Score
Internucleosome distances (bp)
D
s2cell.1 Internucleosome distances
OR_s10.1 Internucleosome Distances (bp)
